# With a Hint of Sudachi: Food Plating Can Facilitate the Fondness of Food

**DOI:** 10.3389/fpsyg.2021.699218

**Published:** 2021-10-15

**Authors:** Nao Kokaji, Masashi Nakatani

**Affiliations:** ^1^Graduate School of Media and Governance, Keio University Shonan Fujisawa Campus, Fujisawa, Japan; ^2^Faculty of Environment and Information Studies, Keio University Shonan Fujisawa Campus, Fujisawa, Japan

**Keywords:** visual perception, food pairing, food plating, image analysis, sensory science

## Abstract

Among the senses of food, our subjective sense of taste is significantly influenced by our visual perception. In appetite science, previous research has reported that when we estimate quality in daily life, we rely considerably on visual information. This study focused on the multimodal mental imagery evoked by the visual information of food served on a plate and examined the effect of the peripheral visual information of garnish on the sensory impression of the main dish. A sensory evaluation experiment was conducted to evaluate the impressions of food photographs, and multivariate analysis was used to structure sensory values. It was found that the appearance of the garnish placed on the plates close to the main dish contributes to visual appetite stimulants. It is evident that color, moisture, and taste (sourness and spiciness) play a major role in the acceptability of food. To stimulate one’s appetite, it is important to make the main dish appear warm. These results can be used to modulate the eating experience and stimulate appetite. Applying these results to meals can improve the dining experience by superimposing visual information with augmented reality technology or by presenting real appropriate garnishes.

## Introduction

Visual information on food significantly influenced our daily life. For example, Arce-Lopera et al. (2012) suggested that luminance distribution affects the judgment of freshness perception of strawberries; Murakoshi et al. (2013) further showed that changes in luminance distribution and visual freshness perception correlate with degradation time even when there are individual differences in foods. These studies reported a significant relationship between freshness and the desire to eat.

The sense of sight plays a major role in determining what consumers find attractive (Schifferstein et al., 2020). For instance, when food is presented in a more pleasant way, people enjoy the food on the plate more (Zellner et al., 2014). Zellner et al. (2011) have also shown that neatly served foods are preferred over foods that are served less neatly.

Previous food studies have investigated how appetite can be aroused not only by the sense of taste but also by the sound, color, and shape of the plate (Roballey et al., 1985; Harrar and Spence, 2013). For example, increasing the visual contrast on a plate (e.g., changing the color of the plate) has been reported to significantly increase food and drink intake in patients with Alzheimer’s disease (Dunne et al., 2004). Using plates and glasses that contrasted with food increased food consumption by 25% and beverage consumption by 84% (Dunne et al., 2004). Furthermore, human-food interaction research is conducted on the eating experience by manipulating the appearance of food. In particular, XR technology can be used, such as augmented reality (AR), virtual reality (VR), and projection mapping (Narumi et al., 2011, 2012; Suzuki et al., 2021). The results of those studies indicate that XR technology has the potential to change consumer behavior and dining experiences.

Main dishes are dishes whose main ingredients are sources of protein such as meat, fish and soybeans, which play an important role in a balanced diet of nutrients, including the supply of high-quality protein (Gabriel et al., 2018). The main dish used in this experiment was fried chicken (Karaage in Japanese), whose garnishing ingredients are frequently placed next to the main dish. This is the national dish of Japan. Deep-fried dishes, such as fried chicken, contain fat (Marangoni et al., 2015), which should be consumed by elderly people and can provide a large amount of energy in a small quantity.

Garnishes often play completely different roles in different cultures and dishes and can be widely discussed in terms of colors, shapes, numbers, quantities, sizes, varieties, arrangements, flavors, tastes, satiety, and aromas (McCrickerd et al., 2012; Carney et al., 2018; Spencer and Guinard, 2018). Some ingredients are intended to be eaten, while others are mainly used to add color to a dish. In this paper, we define “garnish” as a solid food. Liquid condiments such as mayonnaise and other sources were excluded from this study. Garnishes on the plates were not determined by the number of pieces but by the amount of garnish that would fill the blank space next to the main dish.

There have been studies of visual appetite stimulants and surrounding colors and other factors; however, few cases have investigated the significance of the immediate element of the surrounding environment of the main dish: the garnish. Therefore, this study tested the effect of peripheral visual information, garnish, on the sensory impression of the main dish. We conducted a sensory evaluation experiment to study the impressions of food photographs and conducted multivariate analysis to exploratorily structure the sensory values.

In this study, we conducted an experiment to find the structural model of appetite stimulants. This study focused on the multimodal mental imagery evoked by the visual information of food served on a plate and examined the effect of the peripheral visual information of garnish on the sensory impression of the main dish. When looking at the visual stimulus images, we speculated that not only the color obtained from the visual information but also the freshness of the food and the impression of dryness and wetness estimated by looking at the images might affect the stimulated appetite for the main dish. In this article, we use the term “visual appetite stimulants” to describe the effect of appetite increase when images of a dish are observed. This study clarifies the possibility that even the combination of a garnish and a main dish, which is not intended to be eaten or has never been experienced, can change the stimulated appetite for the main dish.

This research contributes to the field of investigating the relationship between material perception via vision and perception of food/eating behavior. Although research on estimating the caloric value of food in images taken with mobile devices has been widely conducted (Domhardt et al., 2015; Dinic and Stutz, 2017; Fang et al., 2019), research on the relationship between visual appetite stimulants and garnishes and information superposition has rarely been investigated. Additionally, the key findings from this study are useful for consumer services to optimize food purchasing decisions based solely on appearance when purchasing lunchboxes or prepared foods at supermarkets or delicatessens or by looking at photographs of dishes on the menu or online delivery. In these situations, consumers are likely to make judgments based on past experience and food appearance. This research is also helpful for those who are suffering from eating disorders.

## Materials and Methods

### Participants

The study participants included 15 students (11 women and 4 men; mean age, 22.0 years; SD, 1.37 years; age range, 21–26 years). Each participant had normal color vision and normal or corrected-to-normal visual acuity. The SFC Research Ethics Committee on Human Experimentation of Keio University, Shonan Fujisawa Campus (approval # 302), approved the experimental protocol. Informed consent, in writing, was collected from each participant prior to the experiment.

### Stimuli

**FIGURE 1:**
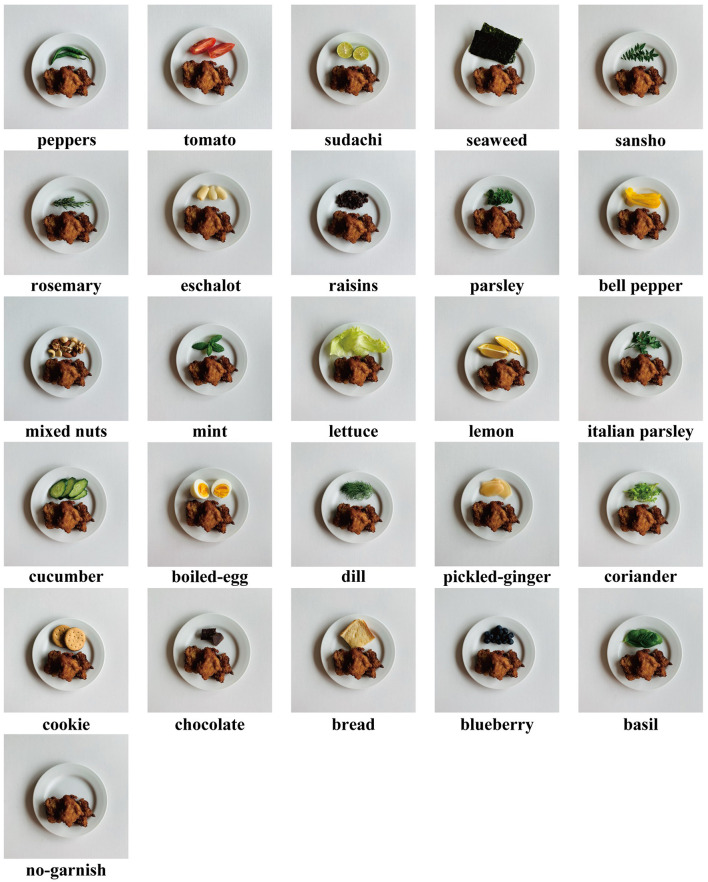
Visual stimuli used in sensory evaluation experiments. We created 26 types of stimuli, including the no-garnish plate: unchanged plates and main dish (fried chicken). The ingredients were selected based on the results of a 10-participant pilot study with 30 types of garnish. Of these, 25 were chosen from the perspective of color, taste, and nutrition.

We created 26 types of stimuli, including the no-garnish plate: unchanged plates with one main dish (fried chicken) (Figure 1). The 25 types of garnishes included ingredients such as lemon and parsley, which are commonly used for garnishing. Additionally, we used seaweed, eschalot (rakkyo in Japanese), and sweet ingredients, which are uncommonly used. The ingredients were selected based on the results of a 10-participant pilot study with 30 types of garnishes. Of these garnishes, 25 were chosen from the perspective of color, nutritional balance, crop classification (leafy vegetables, fruits, etc.) and flavor. The ingredients excluded from the results of the preliminary study included seaweed, potato chips, corn, mushrooms, kimchi, pickles (gherkins), white rice, crème fraîche, and fried noodles.

### Procedures

Participants evaluated each of the 26 images using a visual analog scale (VAS). The 11 adjective pairs are presented below. The order of the stimuli was randomized across participants, and the participants could view the reference images at any time during the experiment. The participants provided their evaluations on a full-screen browser using Google Forms (Google LLC).

The eleven adjective pairs used in sensory evaluation were as follows:

• Freshness/fresh – not fresh
• Drying/dry – not dry
• Wetness/wet – not wet
• Warmth/warm – not warm
• Sourness/sour – not sour
• Sweetness/sweet – not sweet
• Saltiness/salty – not salty
• Looking/nice-looking – nasty looking
• Fondness/like – dislike
• Deliciousness/tasty – not tasty
• Appetite/want to eat – do not want to eat

### Statistical Analysis

#### Factor Analysis

We analyzed the eleven abovementioned adjective pairs. Factor analysis employed the principal component method and varimax rotation. For multivariate analysis, we used Microsoft Excel (Version 16.44) and SPSS Statistics (Version 26.0.0.1). The data were collected using Google Forms (Google LLC) and organized. The mean values were calculated using Excel.

#### Multiple Regression Analysis

We applied multiple regression analysis to the values and excluded “looking,” “fondness,” “deliciousness,” and “appetite,” which were positioned as higher-order variables, and we conducted multiple regression analysis using the factor scores.

#### Principal Component Analysis

We also conducted principal component analysis of the covariance matrix without rotation.

#### Cluster Analysis

To clarify how the types of garnish were classified, we conducted cluster analysis (Ward Linkage) using the principal component scores of principal component 1 (PC1) and principal component 2 (PC2).

## Results

### Factor Analysis

The factor loadings were 80.7%, and Table 1 lists each factor’s cumulative contribution rates. Factor 1 was the “warm and non-sweet factor.” This factor was affected by the warmth and appearance of the dishes. Appetite and favorability were associated with warmth and good appearance. Factor 2 was the “fresh and refreshing factor,” where wetness, freshness, and sourness simultaneously occurred. These factors affect appetite. Factor 3 was the “saltiness factor.” In the preliminary questionnaires of this experiment, lemon was the first that came to mind when asked about fried chickens’ garnish (Supplementary Table 1). Additionally, we calculated means (standard deviations) and ranking for “appetite” (Supplementary Table 1). Based on factor analysis and the results in Table 1, boiled eggs, bread, cookies, and mixed nuts, which contain little moisture, are less likely to stimulate appetite that foods that are relatively wet. Thus, choosing a moist garnish might stimulate appetite. The factor scores were standardized such that the mean was 0 and the standard deviation was 1 for each factor (Table 1).

**TABLE 1:** The results of factor analysis show the elements that change the impressions of the main dishes from the viewpoint of temperature, taste, and freshness.

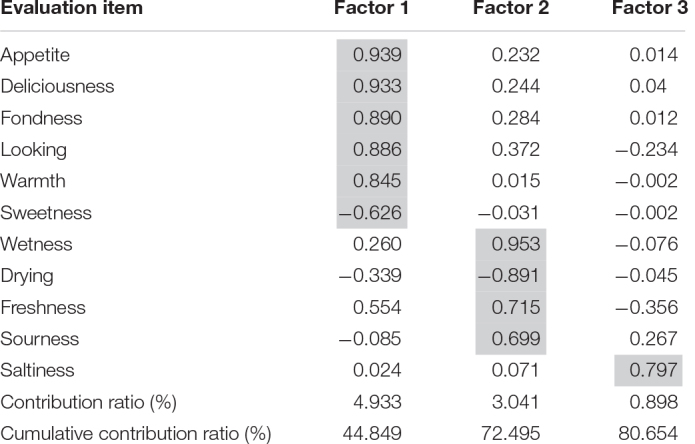

*The factor loading was 80.7%. Factor 1 was affected by the warmth and appearance of dishes. In Factor 2, wetness, freshness, and sourness co-occurred. We named Factor 1 the “warm and non-sweet factor.” Factor 2 was the “fresh and refreshing factor,” and Factor 3 was the “saltiness factor.”*

### Multiple Regression Analysis

**FIGURE 2:**
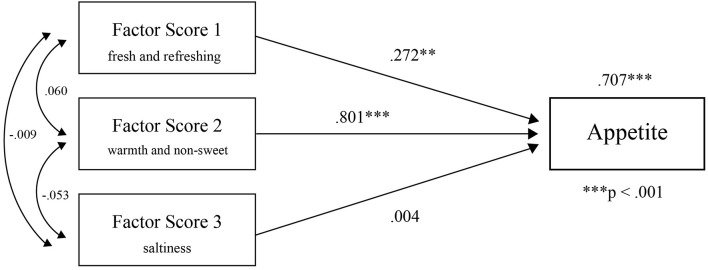
Factor 2 has more influence than Factor 1 on appetite from the high correlation coefficient scores. We named Factor 1 the “fresh and refreshing factor.” Factor 2 was the “warm and non-sweet factor,” and Factor 3 was the “saltiness factor.” ** and *** denotes *P* < 0.01 and *P* < 0.001, respectively.

To investigate the factors that stimulated appetite, we performed multiple regression analysis using the factor scores, which excluded “looking,” “fondness,” “deliciousness,” and “appetite,” (Supplementary Table 2), as they were positioned as higher-order variables (Table 2). Since this research is an exploratory study, we positioned “looking,” “fondness,” “deliciousness,” and “appetite” as higher-order variables compared to “freshness,” “drying,” “wetness,” “warmth,” “sourness,” “sweetness,” and “saltiness,” which are related to impressions received from taste, temperature, and appearance. We decided to perform multiple regression analysis by first separating the variables related to the sensation itself (sour, dry, wet, etc.) and the overall evaluation variables (stimulation of appetite as a result, etc.). Each of variance inflation factor (VIF) obtained in the analysis was less than 10 (Table 3). This result is shown in the path diagram in Figure 2. According to these analyses, Factor 1 and 2 scores had a statistically significant effect on appetite. Moreover, Factor 2 had a greater influence on appetite than Factor 1 based on the high correlation coefficient scores. Thus, the effects of temperature and taste on the balance of flavors were significantly related to appetite.

**TABLE 2:** We performed factor analysis excluding “looking,” “fondness,” “deliciousness,” and “appetite” for multiple regression.

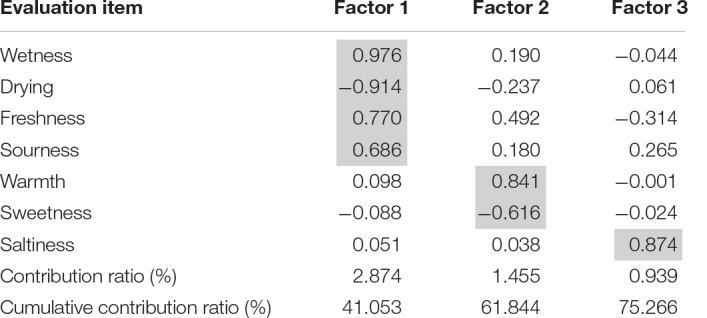

**TABLE 3:** Multiple regression analysis shows that Factor 1 and 2 scores had a statistically significant effect on appetite.

	**Standardized coefficient**	***t***	**Sig.**	**VIF**
Appetite (constant)		55.561	<0.001	
Factor Score 1	0.272	2.512	0.02	1.004
Factor Score 2	0.801	7.382	<0.001	1.006
Factor Score 3	0.004	0.033	0.974	1.003

*Standardized coefficient, *t*-test value and significance, and collinearity statistics for each factor are listed. In this analysis, All of VIFs < 10 were obtained. The Factor 2 standardized coefficient was 0.80.*

### Principal Component Analysis

Principal component analysis was conducted on all 11 variables divided into two groups: physical and sensitivity factors. The principal component loadings were analyzed by the principal components within the variance-covariance matrix.

*Physical factors: dryness/wetness/warmth/sourness/sweetness/saltiness*

Two main principal components were identified (Table 4). PC1 extracted “moisture,” “sourness,” and “saltiness,” while PC2 extracted “sweetness” and “coolness.” We named PC1 the “complementary moisturization and refreshment component,” and PC2 was the “strong influence of sweet garnish.”

**TABLE 4:** Result of principal component analysis: principal component loadings of six adjective pairs.

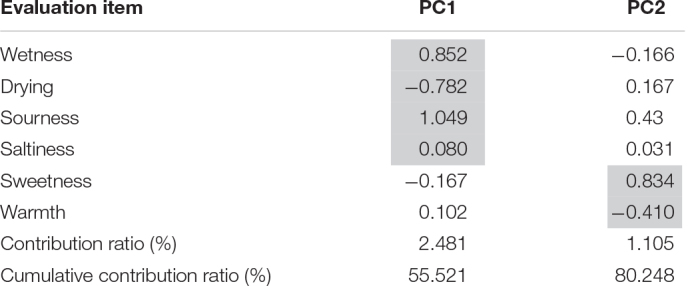

*PC1 extracted “moisture,” “sourness,” and “non-saltiness,” while PC2 extracted “sweetness” and “non-warmth.” Results of principal component analysis except fondness/appetite/deliciousness/looking/freshness. We named PC1 the “complementary moisturization and refreshment component,” and PC2 the “the strong influence of sweet garnish.”*

*Sensitivity factors: freshness/deliciousness/looking/fondness/appetite*

Only PC1 was extracted from the sensitivity factors (Table 5). PC1 contained “good looking,” “appetite,” “delicious appearance,” “palatability,” and “freshness.” Therefore, PC1 indicates “freshness from the kitchen.”

**TABLE 5:** Results of principal component analysis except wetness/dryness/sourness/saltiness/sweetness/warmth.

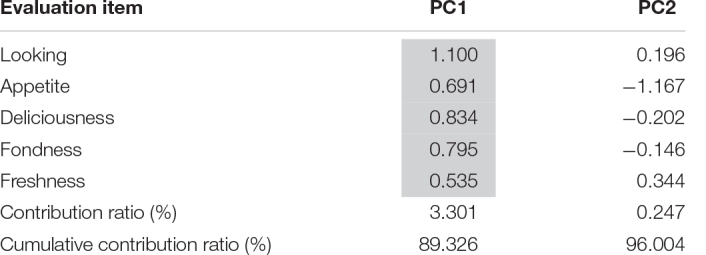

*PC1 extracted “good appearance,” “appetite arousing,” “delicious,” “fond,” and “fresh.” We named PC1 “fresh from the kitchen.”*

*Physical factors and sensitivity factors: drying/wetness/warmth/sourness/sweetness/saltiness/freshness/deliciousness/looking/fondness/appetite*

We conducted principal component analysis of the physical and sensitivity factors (Table 6). PC1 extracted “good appearance,” “delicious,” “fond,” “appetite,” and “non-sweet,” while PC2 extracted “sourness” and “saltiness.”

**TABLE 6:** Result of principal component analysis: principal component loadings of all eleven adjective pairs.

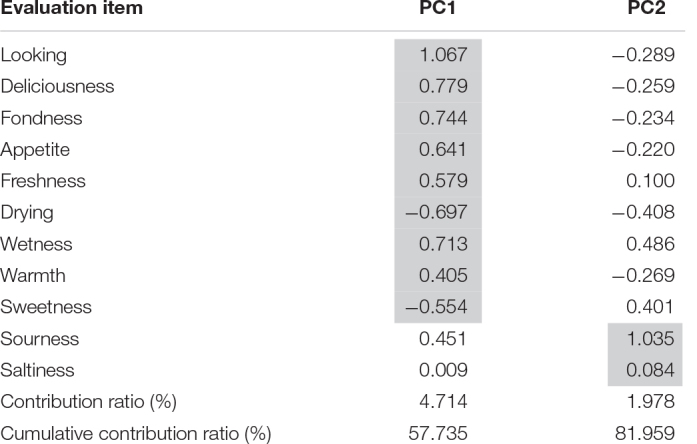

*PC1 extracted “good appearance,” “delicious,” “fond,” “appetite,” and “non-sweet,” while PC2 extracted “sourness” and “saltiness.” PC1 can be regarded as representing “fresh from the kitchen,” and PC2 represents “balance of flavors.”*

The number of axes of the principal components was determined by referring to the scree plot (the horizontal axis represents the principal components, and the vertical axis represents the eigenvalues) (Supplementary Figure 1). The cumulative contribution of PC2 accounted for 82.0% of the total contribution, which exceeded 80%. From this, the number of axes in the principal component was determined to be two. PC1 can be regarded as representing “freshness from the kitchen,” while PC2 represents “balance of flavors.” Principal component scores were calculated. The data were standardized so that the mean was 0 and the standard deviation was 1 for each factor (Table 6).

**FIGURE 3:**
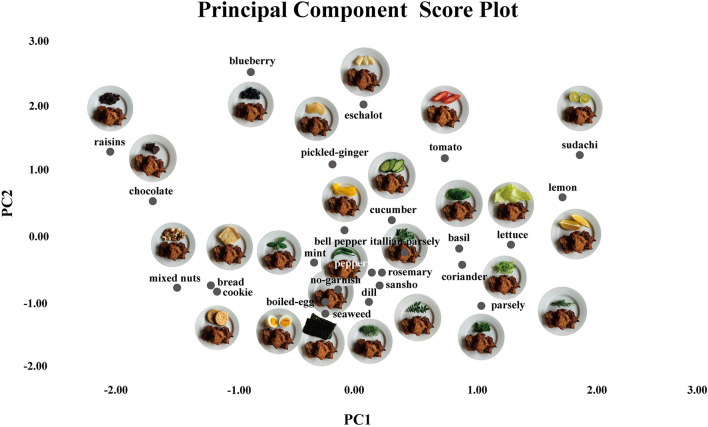
We created a scatter plot based on the principal component scores and superimposed the images of the main dish and the garnish on a two-dimensional plane to visualize the relationship between the main dish and the garnish. Similar-colored garnishes were located close together in this plot. The results of the sensory evaluation showed that sudachi (citrus fruit) was the most suitable garnish to balance the taste of the main dish.

Among the types of garnish, sudachi (citrus fruit) was found to be the most suitable for balancing the taste of the main dish (fried chicken) and making it look like it was freshly prepared. Lemon was found to be a suitable garnish for the main dish. Furthermore, we created a scatter plot based on the principal component scores and superimposed the images of the main dish and the garnish on the two-dimensional plane to visualize the relationship between the main dish and garnish (Figure 3).

### Cluster Analysis

**FIGURE 4:**
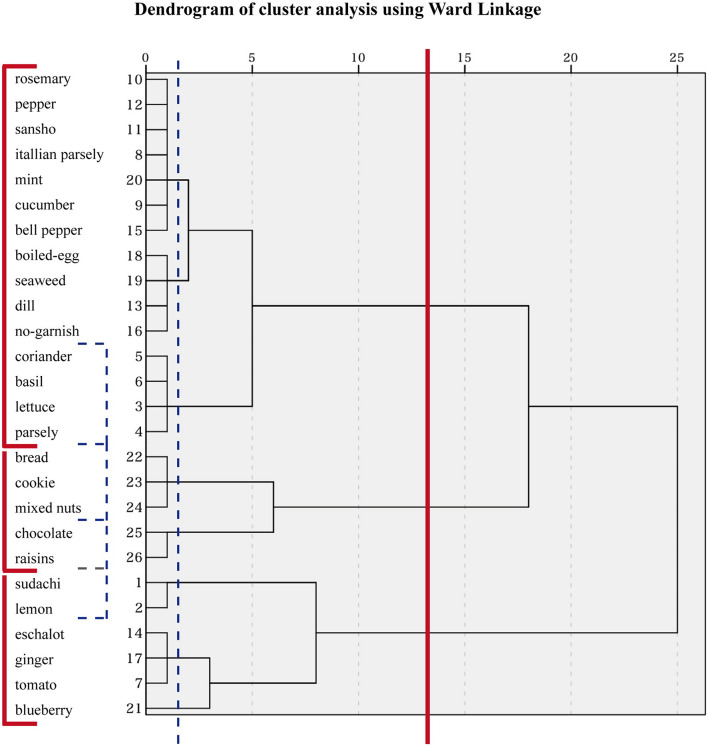
Cluster analysis classified the 26 types of garnish into 3 clusters. The solid line indicates three main clusters, and the dashed line shows more precise clusters in this figure.

Figure 4 shows the dendrogram of the cluster analysis (Ward Linkage) using principal component scores of PC1 and PC2. The dendrogram classifies the 26 types of garnishes into three main clusters: “color,” “dryness and taste,” and “moisture and taste.” In addition, coriander/basil/lettuce/parsley fell under the green vegetable cluster that we named the “green cluster”, and the bread/cookies/mixed nuts cluster and chocolate/raisins cluster were in the “dry and sweet cluster.” The sudachi/lemon cluster was named the “moist and sour cluster.” Thus, the three main clusters included the “green cluster;” “sweet, sour, and spice cluster,” and “wet and dry cluster.”

## Discussion

### Garnishes Can Work as Appetite Stimulants

Multivariate analysis revealed four elements that changed the participants’ impressions of the main dishes. The factor and cluster analyses showed that color, moisture, and taste (sourness and spiciness) play a major role in the impressions of main dishes. Visual attributes, such as the color of the plate, affected the sweetness intensity, perceived flavor, and overall preference of the dish. A study by Piqueras-Fiszman et al., 2013 showed that a strawberry-flavored mousse served on a white plate was perceived as 15% more intense, 10% sweeter, and was 10% more liked when compared to the same dessert served on a black plate. Evidence for physiological changes in the food because of the color, shape, and material of the tableware, such as the plate and cutlery, is also provided by Spence et al. (2012). This study shows that not only the main dish but also the food plating and cutlery can modulate an eating experience. In this research, multiple regression and principal component analyses indicated that to stimulate one’s appetite, it is important to make the main dish appear warm. This indicates that even if the main food is not actually warm, garnish gives observers an impression of warmness in the main dish. It is also plausible that the effect of the color of the ingredients may increase the perception of warmth. Additionally, sour and moist garnishes can affect the appetite because they can balance the flavors of the main dish.

**FIGURE 5:**
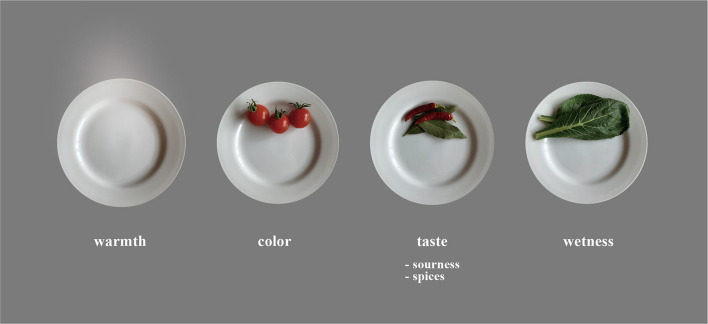
Four representative elements that change the impressions on the main dishes. The results of multivariate analysis showed that color, taste (sourness and spices), and moisture significantly affected the acceptability of a garnish. Additionally, sour and moist types of garnishes balance the taste of the main dish. To stimulate appetite, it is important to make the main dish appear warm, even if it is not fresh.

The multivariate analysis results can be linked to the development of methods implemented to modulate eating experiences and stimulate appetite. For fried foods (e.g., fried chicken), we found ways in which the appearance of the meal contributes to the stimulation of one’s appetite. The current research methodology can be further applied in studies of not only fried food but also boiled, grilled, stir-fried, and steamed food. Methods in which the appearance of a meal contributes to visual appetite stimulants and the design guidelines for meals can contribute to making meals more attractive through the superimposition of information with XR technology, such as AR and VR, or by presenting real objects (Figure 5). Previous research reported that the standard deviation of the luminance distribution of food images influences the perceived visual texture and flavor experience by using AR (Ueda et al., 2020). The development of a real-time modification system without an AR marker could be applied to modify garnishes or the color of dishes. We consider that smart glasses can be applicable for AR of food affection. For example, updated versions of Google Glass and Microsoft Hololens were released in 2019. Although these AR smart glasses are still exploratory, several studies have used smart glasses (Jiang et al., 2018; Chicchi Giglioli et al., 2019; Ueda et al., 2020). For food applications, Ueda et al. (2020) used the method with headgear without AR markers. Jiang et al. (2018) also reported a real-time system for estimating the nutrition of foods using Google glass. Our results contribute to superimposing images of food in restaurants with AR technology presentation, similar to wearing glasses. Especially under the spread of COVID-19, at-home eating has been increasing, so we expect that the mental burden of wearing AR/MR glasses or smart glasses while eating will decrease. Moreover, some people are willing to adapt to information technology while dining alone.

Visual appeal and presentation of a dish can modulate the appetite of those eating it. Several studies have pointed out the importance of the visual presentation of food (i.e., food plating) in the perception of appetite (Michel et al., 2014). The current study supports the claims from previous research and the importance of a garnish. Our exploratory factor analysis showed the hypothetical model that describes the structural relationship between the induced cross-modal perception from visual presentations of food and appetite. Using structural equation modeling (SEM), we plan to conduct another study to test this hypothesis and quantify the overall structures. Additionally, to generalize our claim, further research should include the analysis of individual differences between participants.

Food choice is not solely determined by visual appearance, but when purchasing lunchboxes or prepared foods at supermarkets or delicatessens or by looking at photographs of dishes on the menu or online delivery, consumer mainly choose foods based on food visual appearance. In these situations, we are likely to make judgments based on past experiences and food appearance. Thus, the results of this study will contribute to such situations.

### Limitation and Future Research

Food is closely tied to our past experiences. For example, people who have eaten lemons may taste a sour flavor in their mouths when they see them, but people who have never eaten lemons are unlikely to taste any sourness when they see them. This study takes into account the fact that there is a possibility that people know the taste of each garnish, and the study is based on that assumption. Thus, we interpreted the results as those of having the participants respond to their visual impressions when the participants were presented with the images. However, we did not test the case where the participants observed the garnish for the first time. If unknown material was placed next to the main dish, the participants’ response could be different from that of the current study.

Food garnishes play an important role both explicitly and implicitly. Freshness can be inferred from image statistics of the food (Wada et al., 2010; Arce-Lopera et al., 2012, 2015), and the magnitude of subjective freshness correlates with the parameters of the image statistics (mean, standard deviation, skewness, and kurtosis). In this study, our analysis suggested that the subjective ratings of freshness can influence the appetite of the person for the main food of the dish (Figure 2). If the perception of the garnish placed next to the main food could also affect appetite, we would be able to modulate appetite by changing the visual impressions evoked by the garnish. For example, following our results, we could be garnishing fried chicken (Karaage) with sudachi to stimulate appetite, and garnishing the meal with raisins will decrease appetite. Just as the color of the tableware can change one’s appetite (Dunne et al., 2004), garnishes in the surrounding environment can play a role in getting a person with a small appetite or an eating disorder to eat.

Augmented reality visualization of a served food improves the simulation of the eating process over 3D visualization, which has a positive influence on consumer intention to purchase products (Petit et al., 2021). In the same way, our first impression is visual when buying products or in our desire to eat. Our study possibly contributes to optimizing served foods presented in AR to stimulate consumer appetites.

## Data Availability Statement

The raw data supporting the conclusions of this article will be made available by the authors, without undue reservation.

## Ethics Statement

The studies involving human participants were reviewed and approved by the SFC Research Ethics Committee on Human Experimentation of Keio University, Shonan Fujisawa Campus (approval # 302). The patients/participants provided their written informed consent to participate in this study.

## Author Contributions

NK conducted the experiments, data analysis, and manuscript writing. MN supervised the research project. Both authors conceived and wrote the manuscript.

## Conflict of Interest

The authors declare that the research was conducted in the absence of any commercial or financial relationships that could be construed as a potential conflict of interest.

## Publisher’s Note

All claims expressed in this article are solely those of the authors and do not necessarily represent those of their affiliated organizations, or those of the publisher, the editors and the reviewers. Any product that may be evaluated in this article, or claim that may be made by its manufacturer, is not guaranteed or endorsed by the publisher.
