# Supplementary material for: With a Hint of Sudachi: Food Plating Can Facilitate the Fondness of Food
Source: Front Psychol. 2021 Oct 15;12:699218. doi: 10.3389/fpsyg.2021.699218 (PMC8555489; doi:10.3389/fpsyg.2021.699218)

Supplementary Material

# Supplementary Figures and Tables

## Supplementary Figures
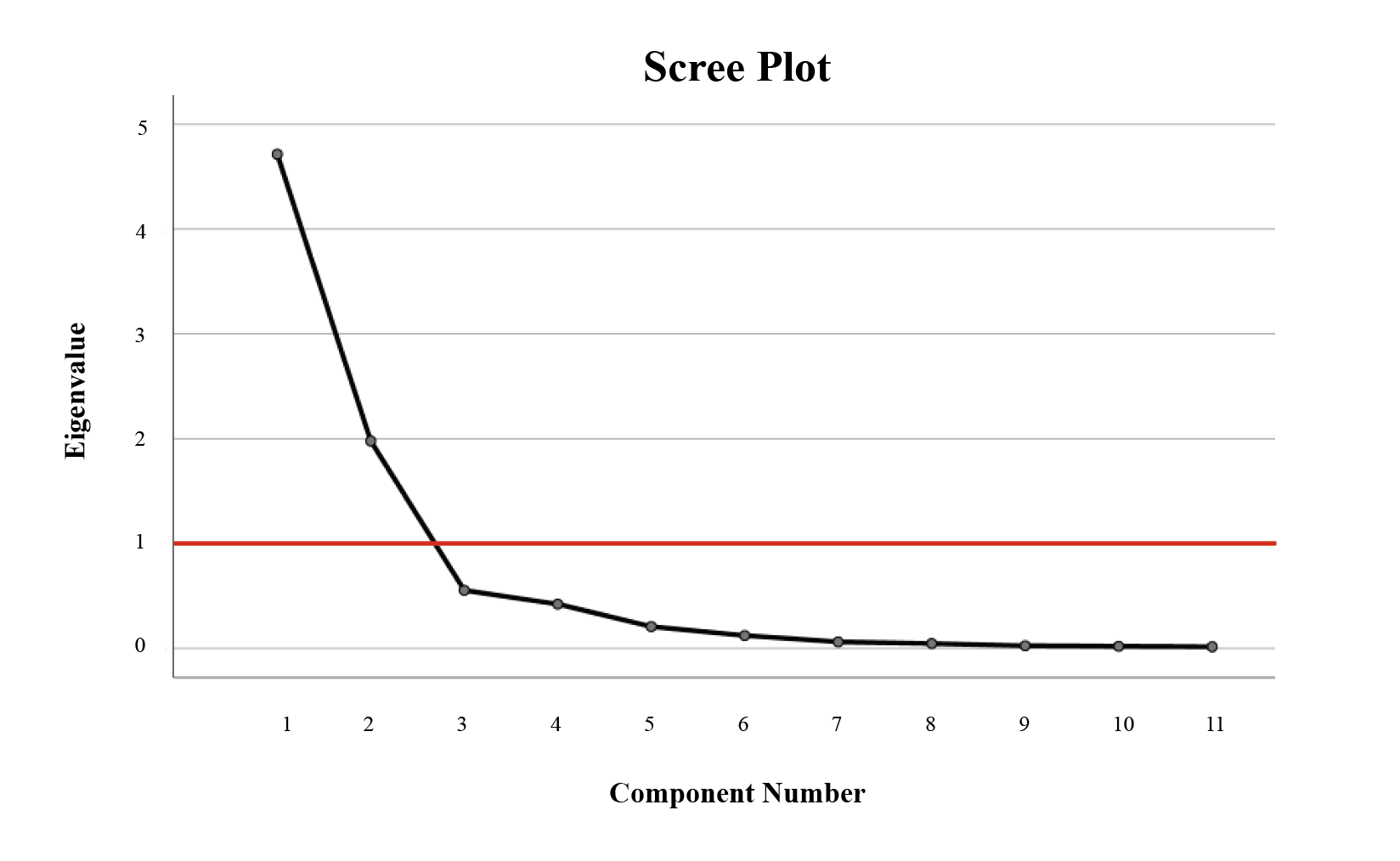


**Supplementary Figure 1.** The number of axes of the principal components was determined by referring to the scree plot (the horizontal axis represents the principal components, and the vertical axis represents the eigenvalues). The cumulative contribution up to PC2 accounted for 82.0 % of the total contribution, indicating that it exceeded 80.0 %.

## Supplementary Tables

**Supplementary Table 1.** The average value (standard deviation) and the rank of the “appetite (want to eat)" for each garnish. In the preliminary questionnaire of this experiment, lemon was also the first thing that came to mind when people were asked about fried chicken. Sudachi was placed as the most suitable one to balance the taste of the main dish (fried chicken) and to make it look like it was more freshly off the kitchen based on the results of multivariate analysis. Based on the results of the factor analysis and the above, we can conclude that it is difficult to arouse the appetite by putting together with boiled eggs, bread, cookies, mixed nuts, etc. because of their dryness.

 
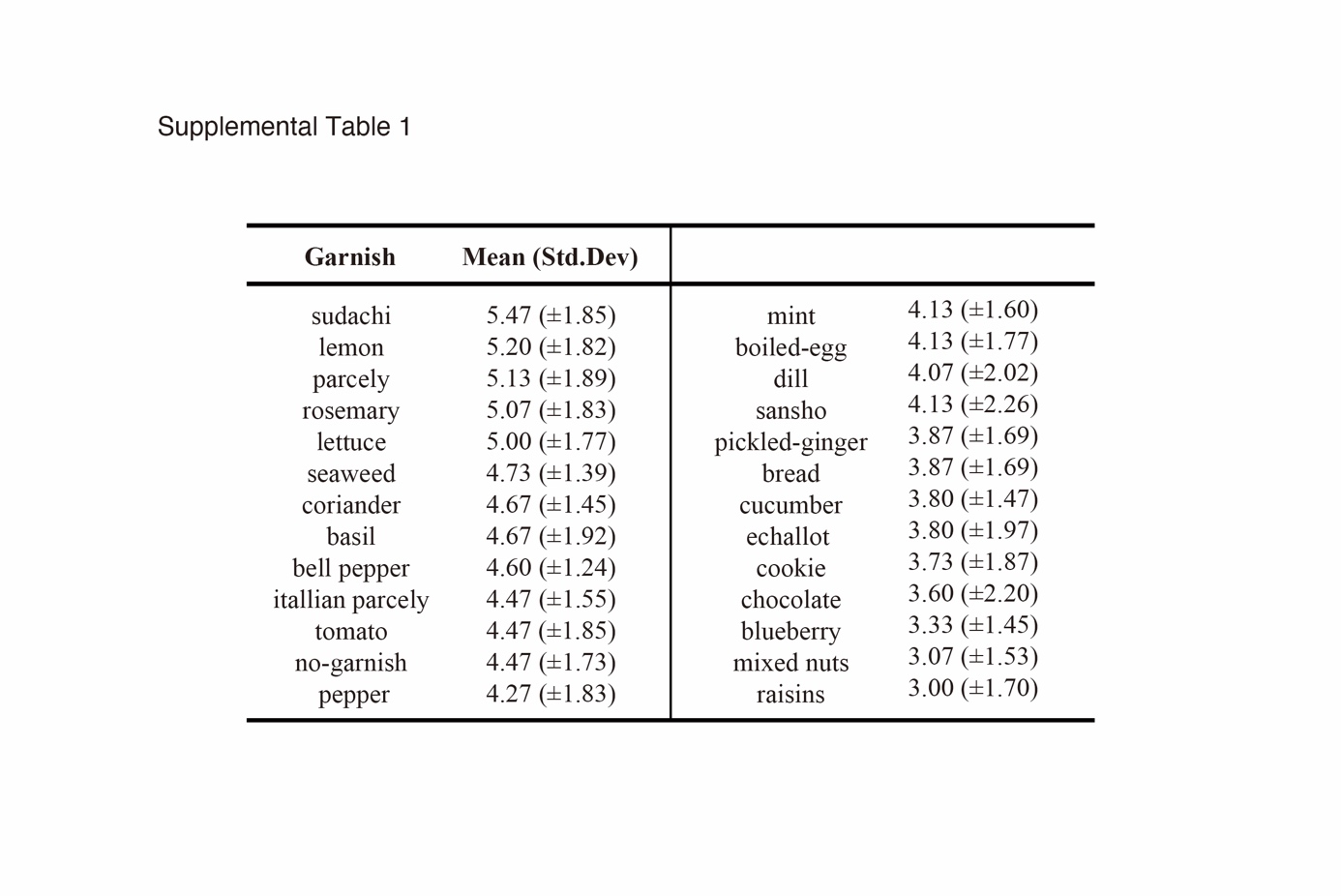


**Supplementary Table 2.** The factor scores, which excluded “looking,” “fondness,” “deliciousness,” and “appetite,” for multiple regression analysis.

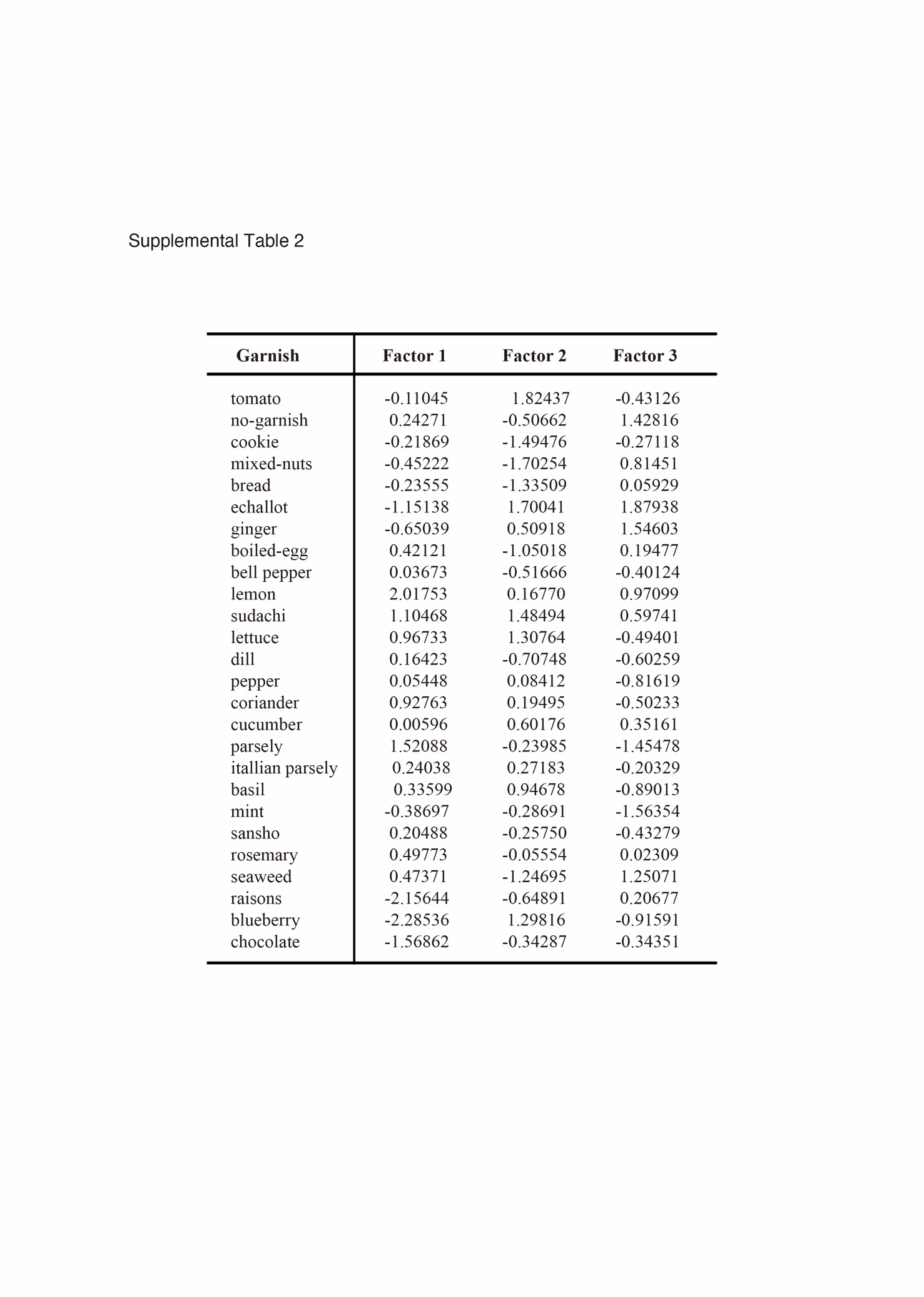

Supplement: Supplementary file 2 [file Data_Sheet_1.docx]
